# ADRD IN CONTEXT: SOCIAL, NEIGHBORHOOD, AND NATIONAL INFLUENCES

**DOI:** 10.1093/geroni/igae098.0043

**Published:** 2024-12-31

**Authors:** Toni Antonucci, Laura Zahodne, Erin Harrell

**Affiliations:** Chair; Co-Chair; Discussant

## Abstract

This symposium showcases innovative work from scholars in the National Institute on Aging (NIA)-funded Alzheimer’s Disease Resource Center for Minority Aging Research, the Michigan Center for Contextual Factors in Alzheimer’s Disease. Each paper examines ecological contexts (e.g., interpersonal relationships, neighborhoods, countries) that influence the experiences of dementia in diverse populations. Dr. Sol and colleagues examine how social resources differentially relate to cognitive health among Black and White older adults living in different types of neighborhoods. Dr. Roche-Dean et al. consider social factors that influence dementia diagnosis awareness across race/ethnicity in a national sample. Although a number of factors did not influence dementia diagnosis awareness in the aggregate sample, analyses stratified by race indicate that married Black participants had greater dementia diagnosis awareness. Dr. Alhasan and colleagues examine community livability and dementia in different South Carolinian counties. Dementia prevalence was higher in less livable counties among women but lower among men. Finally, Dr. Lopez-Anuarbe and colleagues explore differences in health care costs and dementia status among Mexicans and Mexican Americans. They find opposing patterns of association between dementia status and health care costs, as well as contrasting urban-rural differences, among Mexicans in Mexico versus Mexican Americans in the U.S. In sum, each of these papers addresses the critical role of social, neighborhood and national contexts across the dementia continuum and additionally considers variation across sociodemographic groups. Finally, Dr. Erin Harrell from the NIA will offer perspectives on how examining contextual factors can advance NIA’s goal of understanding and eliminating ADRD inequalities.

